# Editorial: Interventions based on new technologies for the management of mood disorders

**DOI:** 10.3389/fpsyt.2022.1099947

**Published:** 2022-12-07

**Authors:** Alejandro Porras-Segovia, Inmaculada Peñuelas-Calvo, Benedicte Nobile, Luis Gutiérrez-Rojas

**Affiliations:** ^1^Mental Health Research Group, Health Research Institute Foundation Jimenez Diaz (IIS-FJD), Madrid, Spain; ^2^Division of Psychiatry, Department of Brain Sciences, Faculty of Medicine, Imperial College London, London, United Kingdom; ^3^Department of Psychiatry, Hospital 12 de Octubre, Madrid, Spain; ^4^INSERM, Montpellier, France; ^5^Department of Psychiatry, University of Granada, Granada, Spain

**Keywords:** new technologies, mental health, mobile applications, social networks, machine learning

Mood disorders are highly prevalent mental conditions that cause significant disability worldwide (1). They are strongly associated with physical comorbidities, such as cancer and cardiovascular diseases, and with other mental conditions, such as anxiety disorders, substance abuse or increased risk of suicide. They also generate significant direct and indirect costs (2).

Despite advances in psychopharmacological treatment and psychotherapy over the last decades, treating depression remains a challenge in daily clinical practice. There are many patients who do not respond to treatment, and there are physical barriers to access to the available treatments (3). Therefore, it is crucial to invest in the development of complementary management strategies, which may help to improve the prognosis of mood disorders.

New technologies are making their way into the mental health arena (4). Studies show the effectiveness of several different devices as a supplementary therapy in mental problems such as suicide (5–7). Their use has been revalued with the COVID-19 pandemic and its blocking periods, compensating for the disruption of face-to-face visits, and helping to maintain continuity of care in mental health. New technologies can continue to help us thanks to their wide availability and versatility.

The goal of this Research Topic is to present studies on therapeutic interventions delivered using new technologies for the management of mood disorders. The potential of these treatments will be discussed, as well as the barriers to their implementation in clinical practice and the developments that are expected in the not distant future.

This Research Topic focus on fronts as diverse as smartphone apps, advances in neuroimaging, transcranial magnetic stimulation, or the use of machine learning.

This Research Topic includes five studies on the use of new technologies and other related devices, systems and techniques applied to the management of mood disorders.

The first of these studies presents the results of the MeMo study, which involves the implementation of a mood monitoring system in young people using a mobile phone application. The authors showed that the use of the app decreased negative mood among participants (Dubad et al.).

The second study consists of an analysis of the publications on the social network Twitter about psychotherapy over the last 11 years in the United States. The authors show that the type of psychotherapy that appears most frequently on this social network is mindfulness, while certain psychotherapies, such as computer-supported therapy, do not appear on the social network, which could be a reflection of the lack of knowledge of these psychotherapy modalities among the general population (Alvarez-Mon et al.).

The third study employs machine learning techniques to detect symptoms of depression and suicide risk through texts extracted from clinical interviews. The authors compared a group of depressed patients with a group of healthy patients and recorded their speech while they responded to a Mini-International Neuropsychiatric Interview. Using machine learning they were able to correctly classify the sample into depressed and non-depressed groups with an area under the curve of 0.9. The classification of suicide risk between high and low within the depressed patients was achieved with an area under the curve of 0.5 (Shin et al.).

The fourth study published in this Research Topic consists of a clinical trial on the use of Tuina therapy and how the results of this treatment can be reflected in changes in the MRI of depressed patients (Tao et al.).

The fifth study to be published explores the use of bilateral repetitive transcranial magnetic stimulation to tackle sleep disorders and Hypothalamic-pituitary-adrenal (HPA) Axis Dysfunction in Subjects with Major Depression. After 4 weeks of treatment, a beneficial effect was found in those patients treated with the intervention (Chen et al.).

These five studies, each one with its different angle, contribute to the growing evidence that new technologies can help us in the management of mood disorders.

Future perspectives in the field include the development of high-quality software and devices, and more accurate analysis techniques, and how to overcome challenges to the implementation of new technologies in everyday clinical practice.

## Author contributions

AP-S wrote the manuscript. All authors contributed substantially to the drafting and revision of the manuscript, read, and approved the final version.
